# *Toxoplasma gondii* in raccoons (*Procyon lotor*) in Germany: a serosurvey based on meat juice

**DOI:** 10.1007/s00436-022-07646-w

**Published:** 2022-09-23

**Authors:** Lydia Engel, Ahmad Hamedy, Aleksandra Kornacka-Stackonis, Torsten Langner, Stefan Birka, Martin Koethe

**Affiliations:** 1 Institute of Food Hygiene, Leipzig University, An den Tierkliniken 1, 04103 Leipzig, Germany; 2 Witold Stefański Institute of Parasitology, Polish Academy of Sciences, Twarda 51/55, 00-818 Warsaw, Poland

**Keywords:** Protozoan, Game, ELISA, Tissue fluid, Zoonosis, Seroprevalence

## Abstract

*Toxoplasma gondii* seroprevalence was determined in meat juice samples of 820 free-living raccoons from Germany. The animals were collected between December 2017 and April 2021. Using a commercial enzyme linked immunosorbent assay (ELISA), the overall seroprevalence was found to be 48.5%. Statistical analysis revealed significant seroprevalence differences between seasons, sex, and weight of analysed raccoons. The prevalence in late winter/spring (57.7%) was significantly higher than in autumn (38.4%) (*p* < 0.0003). Male raccoons (50.5%) were more often seropositive than females (41.0%) (*p* = 0.028). Increasing animal weight had a significant impact on the relative probability of a positive serostatus (odds ratio: 1.783, *p* < 0.0001). Furthermore, we found regional differences in seroprevalence, but there was no statistically significant difference resulting from animal age, degree of habitat urbanization and hunting year. Meat juice is a suitable medium for serological surveys for *T. gondii* in meat producing animals, as sampling is even possible after slaughter or during meat inspection when blood is no longer available. The observed high seroprevalence indicates that *T. gondii* infection is widespread among the German raccoon population providing a potentially relevant source of *T. gondii* transmission to humans upon consumption or handling of animal products.

## Introduction

*Toxoplasma gondii* is a ubiquitous intracellular parasite able to infect all warm-blooded animals as well as humans. To date, about one-third of the world population is infected with this protozoan (Dubey 2021). The infection rate may vary on a national basis, e.g. in Germany about 55% of the general population was found to be infected with *T. gondii* (Wilking et al. 2016). While in immunocompetent individuals the infection mostly stays asymptomatic or only causes mild symptoms, in immunocompromised patients it can lead to serious pathological effects (Montoya and Liesenfeld 2004). Additionally, there is a high risk to the foetus if a seronegative mother becomes infected during pregnancy, primary infection can lead to abortion or a wide range of other manifestations like encephalitis, pneumonia or chorioretinitis (Jones et al. 2014; Lopes et al. 2007).

There are three infectious stages of *T. gondii*: sporozoites (in oocysts), tachyzoites and bradyzoites. Felidae are the only definitive hosts in which sexual development of the protozoan results in the excretion of oocysts with their faeces. After infection, sporozoites develop into tachyzoites that disseminate within the host and transform into bradyzoites accumulating within tissue cysts in muscles and organs. Besides congenital transmission, humans can get infected by either taking up oocysts from the environment or by ingestion of raw or undercooked meat containing tissue cysts (Montoya and Liesenfeld 2004). Approximately 50% of *T. gondii* infections in the USA are assumed to be foodborne, making *T. gondii* one of the most important foodborne pathogens in the USA (Scallan et al. 2011). In Europe, the consumption of raw or undercooked meat and cured meat products, including game, is the most important source of infection (Baril et al. 1999; Cook et al. 2000). Game meat in particular was supposed to be the cause of some severe toxoplasmosis cases (Carme et al. 2002; Ross et al. 2001). Furthermore, McDonald et al. (1990) have shown that consumption and handling of game meat as well as frequency of consumption was significantly associated with an infection of Canadian pregnant women. There are also published cases of an acute toxoplasmosis outbreak in hunters due to consumption of game meat in the USA (Sacks et al. 1983). As hunters consume game meat more frequently, they are considered a high-risk group in respect of getting in contact with meat containing *T. gondii* tissue cysts, as revealed in a study in Slovakia, recently (Fecková et al. 2020).

The raccoon (*Procyon lotor*) was originally native in North America and has established in Germany since the early twentieth century as an invasive species (Beltrán-Beck et al. 2012). Due to their omnivorous feeding habits, raccoons are a good indicator of zoonoses or environmental contamination and act as sentinel hosts for *T. gondii* (Bigler et al. 1975; Dubey 2021). According to the German Hunting Association (DJV), the consumption of game meat is becoming increasingly popular (German Hunting Association 2019). The annual hunting numbers of raccoons in Germany have risen sharply in recent years, from about 8000 in 1999/2000 to more than 200,000 in 2019/2020 (German Hunting Association 2021). Although consumption of raccoon meat is rare in Germany, the increasing number of hunted animals may raise interest in use of their meat for human consumption. From the USA, it is known that raccoon meat is consumed, although to a lesser extent than other game animals and also that hunters do eat raccoon meat more often than non-hunters (Burger 2000; Gaines et al. 2000; Goguen and Riley 2020).

There is some data on the prevalence of *T. gondii* antibodies in raccoons for several regions of the world. Mostly, they are based on examinations of blood. Based on this sample material, in North America seroprevalence ranges from 13 to 84.4% (Burridge et al. 1979; Gerhold et al. 2017; Hancock et al. 2005; Hill et al. 1998; Hwang et al. 2007; Mitchell et al. 1999; Smith and Frenkel 1995). In Japan, Sato et al. (2011) found a seroprevalence of 9.9% in feral raccoons. In Germany, the seroprevalence was previously reported to be about 37.4% (Heddergott et al. 2017). However, blood is not an optimal sample material to examine game animals after hunting because it is only available directly after killing. Meat juice can be obtained for a long period of time, e.g. by freezing and thawing, and therefore is more suitable for examination of hunted game animals for the presence of antibodies. The benefit is that meat juice can still be taken at meat inspection, after the carcass has been frozen, or even if there are only carcass parts available. But, there is only one study focusing on detection of *T. gondii* antibodies in raccoons based on meat juice. This study only comprised a small sample size (*n* = 12) and was limited to one study area in Germany, the federal state Mecklenburg-Western Pomerania. Thus, the reported antibody prevalence of 33.3% may be of limited precision (Kornacka et al. 2018). The aim of the present study was to gain information on a large sample of the German raccoon population about the presence of *T. gondii* antibodies in meat juice and on respective influencing factors. This data is compared with previous results to estimate the value of meat juice results and to better assess the potential public health risk posed by raccoon meat.

## Material and methods

### Sample collection and information

A total of 820 raccoons were sampled in a German fur producing company (Fellwechsel Vertriebs GmbH, Löptin, Germany). These animals’ carcasses were previously collected by Fellwechsel GmbH after being killed by local hunters or, in few cases, after having been killed in accidents. They originate from the four hunting seasons 2017/2018, 2018/2019, 2019/2020, and 2020/2021. The carcasses were stored constantly frozen until being used for fur production at − 18 °C. Defrosted carcasses were sampled after skinning. Each carcass was individually labelled and assigned to corresponding accompanying information, which comprised hunting date and origin (German postcode) of the animals. Sex, age, and weight of the animals were determined during sample collection. Age was classified into adult and juvenile based on deciduous or permanent dentition as originally described by Grau et al. (1970) and recently applied by Hwang et al. (2007).

Based on the given postcodes, the animals’ origin was further assigned to a specific Federal State and Local Administrative Unit (LAU) using official data from the German Federal Statistical Office (Statistisches Bundesamt 2021). This data also contains the degree of urbanization for every German LAU based on the classification developed in 2011 by DG AGRI and DG REGIO of the European Commission with support of the Joint Research Centre (JRC) and Eurostat (Eurostat 2011). This classification distinguishes densely populated areas (cities and large urban areas; coded as 1) from intermediate density areas (towns and suburbs, and small urban areas; coded as 2), and thinly populated areas (rural areas; coded as 3). In some cases, postcodes comprised several LAUs or even Federal States. In these cases, urbanization classification was manually assigned to a specific code when all respective LAUs belonged to the same code. When LAUs of different urbanization degrees were part of a common postcode, an intermediate code was computed (i.e. 2.5 for comprised LAUs of code 2 and 3).

Missing information: For some animals, not all accompanying information was available. Postcodes were missing for 113 and hunting dates for 123 raccoons. Due to the carcasses’ condition, definite sex determination was impossible for eleven animals, while weight measurement of two animals and age determination for another two animals could not be performed.

From each carcass, the following samples were collected for further examination: head, one forelimb, flexor muscles of the other forelimb, one hind limb, gastrocnemius muscle of the other hind limb, diaphragm and in some cases back musculature. All samples of an individual animal were stored together in a plastic bag. These samples were frozen at − 24 °C onsite and transported frozen to the Leipzig University, Institute of Food Hygiene. They were kept frozen until further examination.

### Serology

Serological examination was performed on meat juice. Samples were thawed 2 days in a refrigeration chamber at 1 °C. Meat juice was collected out of every bag with a sterile 10 ml syringe into a sterile sample vessel and stored frozen at − 19 °C (± 2 °C) until used for testing. The presence of antibodies against *T. gondii* was determined for every meat juice sample using a commercial indirect ELISA (ID Screen Toxoplasmosis Indirect Multi-species, IDvet, France) according to the most recent manufacturer’s instructions for meat juice (50 µl sample volume at 1:2 dilution). This ELISA has been successfully applied to raccoon meat juice samples by others, recently (Kornacka et al. 2018). Optical density was measured at 450 nm using a microplate reader (Tecan Infinite F50, Germany). Using internal positive and negative controls, the sample to positive control ratio (S/P ratio) was calculated by using the following formula as indicated in the instructions:

$${~}^{S}\!\left/ \!{~}_{P }\right.\%=\frac{{OD}_{value\;of\;the\;sample}- {OD}_{value\;of\;the\;negative\;control}}{{OD}_{value\;of\;the\;positive\;control}- {OD}_{value\;of\;the\;negative\;control}} \times 100$$

For meat juice, samples with S/P ≤ 25% were considered negative for *T. gondii* antibodies. Samples with an S/P ratio between 25 and 30% were considered doubtful. If the S/P ratio was ≥ 30%, the sample was considered positive for presence of *T. gondii* antibodies. All samples were analysed in duplicates and the mean value was used for result calculation. Samples with a standard deviation of more than 15% between the two replicates were examined repeatedly in case these deviations could have led to a different evaluation of the result. In addition to the assay’s internal positive control, samples of previously positive tested raccoon meat juice were included in each assay. These positive meat juice samples were kindly provided by the Witold Stefański Institute of Parasitology, Polish Academy of Sciences, Warsaw, Poland. To check assay performance for interassay variation one low (S/P about 40%) and one high (S/P about 150%) positive meat juice samples were included in every assay.

### Statistics

All statistical analyses were performed by Prism9 Software (GraphPad Software, LL, USA). Chi-square tests were used for analysis of differences in prevalence of sex, age, urbanization, season, and hunting year. Bonferroni correction was applied for analyses with more than two groups when the global chi-square test revealed statistical significance. Analysis of weight was done using logistic regression for odds ratios and likelihood ratio test. Differences were considered statistically significant when the *p* value was < 0.05.

## Results

The overall seroprevalence of *T. gondii* in examined raccoon meat juice samples was 48.5% (398/820; 95% confidence interval 45.1–52.0). Another 48.5% (398/820; 95% CI: 45.1–52.0) were negative and 2.9% (24/820; 95% CI: 2.0–4.3) of the samples were considered doubtful for the presence of antibodies to *T. gondii*. S/P ratio of positive samples ranged from 30.0 to 292.5%. Further information on S/P ratio distribution is shown in Table 1.

**Table 1:** Sample distribution regarding serostatus based on S/P ratios

< 25%	25–30%	30–60%	60–120%	≥ 120%	Total
Negative	Doubtful	Low positive	Medium positive	High positive	
398	24	103	177	118	820
48.5%	2.9%	12.6%	21.6%	14.4%	100%

Presence of antibodies seems not to be dependent on age, degree of urbanization, and the hunting year because the performed respective chi-square tests revealed no significant differences. However, antibodies were found in 48.8% of adults and 29.4% of juveniles. Regarding the degree of urbanization, 51.6% of animals originating from densely populated areas (Code 1), 48.5% from intermediate density areas (Code 2), 47.2% from thinly populated areas (Code 3) and 52.6% from areas comprising Code 2 and Code 3 areas (Code 2.5) were positive for the presence of *T. gondii* antibodies. The seroprevalence in different hunting years ranged from 46.4 to 56.3% (see details in Table 2).

**Table 2:** Seroprevalence of *T. gondii* in sampled raccoons associated with variables

Variable	Category	No. tested	No. positive	Prevalence [%]	95% CI	*p*-value ^a^
Age						0.113
	Adult	801	391	48.81	45.4–52.3	
	Juvenile	17	5	29.41	13.3–53.1	
	Subtotal	818				
Urbanization ^b^						0.836
	Code 1	62	32	51.61	39.4–63.6	
	Code 2	270	131	48.52	42.6–54.5	
	Code 2.5	57	30	52.63	39.9–65.0	
	Code 3	318	150	47.17	41.7–52.7	
	Subtotal	707				
Hunting year ^c^						0.809
	2017/2018	16	9	56.25	33.2–76.9	
	2018/2019	181	84	46.41	39.3–53.7	
	2019/2020	247	124	50.20	44.0–56.4	
	2020/2021	253	123	48.62	42.5–54.7	
	Subtotal	697				
Season ^d^						< 0.0003
	Autumn	219	84	38.36	32.2–44.9	
	Winter	138	63	45.65	37.6–54.0	
	Late winter/spring	331	191	57.70	52.3–62.9	
	Subtotal	688				
Sex						0.028
	Male	643	325	50.54	46.7–64.4	
	Female	166	68	40.96	33.8–48.6	
	Subtotal	809				
Total		820	398	48.54	45.1–52.0	

^a^*p*-value of global chi-square test per variable

^b^Code 1 = Cities; Code 2 = Towns and suburbs; Code 2.5 = postal code included local area units of Code 2 and 3; Code 3 = Rural areas

^c^Hunting year runs from April 1st to March 31st of the next year

^d^Autumn = 06 Sep–14 Dec; winter = 15 Dec–14 Jan; late winter/spring = 15 Jan–10 Apr

**Fig. 1:**
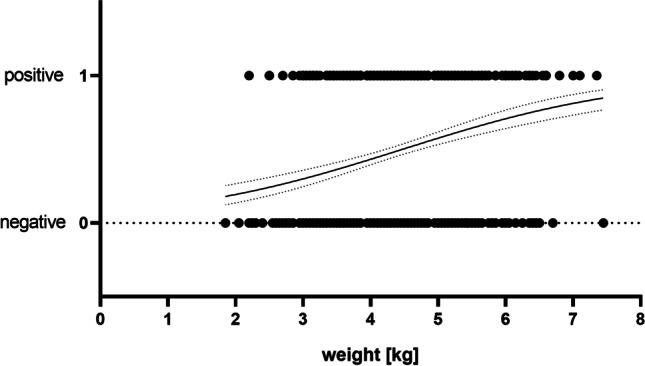
Simple logistic regression of raccoon serostatus in dependence of animal weight (*n* = 818)

Statistically significant differences were found for season, sex and weight. Detailed results are compiled in Table 2. Compared to animals from autumn, raccoons hunted in late winter to spring were statistically significant more often seropositive (57.7% vs. 38.4%, *p* < 0.0003, Bonferroni corrected pairwise chi-square comparisons). Prevalence of animals hunted in winter (45.7%) did not differ significantly from either group. Seroprevalence was significantly higher in male (50.5%) than in female (41.0%) animals (*p* = 0.028, chi-square test). The weight of all raccoons included in this study ranged from 1.85 to 7.45 kg. Analysing animal weight and respective serostatus by simple logistic regression, the relative probability of a positive result was shown to increase by 78.3% per kg (odds ratio [OR]: 1.783, 95% CI: 1.516–2.108; *p* < 0.0001, likelihood ratio test). The graphical result of this analysis is shown in Fig. 1.

**Fig. 2:**
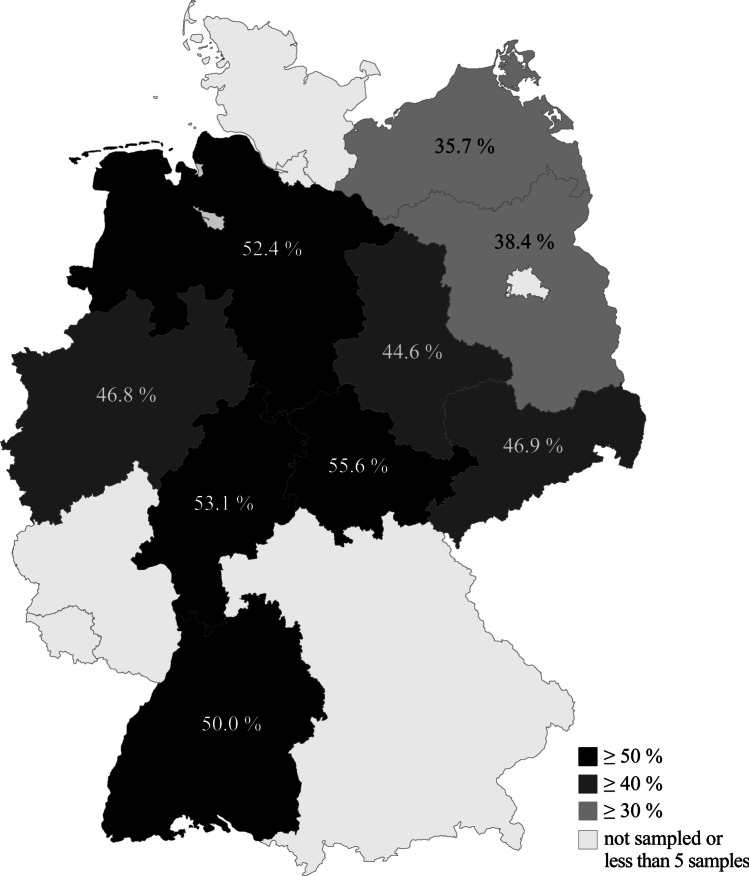
Regional distribution of seroprevalence ranges in Germany; original clean map was created using Microsoft Excel

The prevalence varies greatly among the sampled areas. As shown in Table 3, the comparably highest seroprevalence was determined in the German federal states of Thuringia (55.6%), Hesse (53.1%) and Lower Saxony (52.4%). In contrast, animals from Mecklenburg-Western Pomerania (35.7%) and Brandenburg (38.4%) presented the lowest seroprevalence in Germany (see Fig. 2 for regional distribution). Some deviating prevalence estimates were based on only few samples.

**Table 3:** Seroprevalence in German federal states

German federal state	No. positive/no tested	Seroprevalence [%]	95% CI
Baden-Württemberg	9/18	50.0	29.0–71.0
Bavaria	2/4	50.0	8.9–91.1
Brandenburg	5/13	38.4	17.7–64.5
Hesse	93/175	53.1	45.8–60.4
Lower Saxony	55/105	52.4	42.9–61.7
Mecklenburg-Western Pomerania	5/14	35.7	16.3–61.2
North Rhine-Westphalia	80/171	46.8	39.5–54.3
Rhineland-Palatinate	0/1	0.0	0.0–94.9
Saarland	2/2	100.0	17.8–100.0
Saxony	15/32	46.9	30.9–63.6
Saxony-Anhalt	54/121	44.6	36.1–53.5
Thuringia	21/37	55.6	39.6–70.5

## Discussion

Studies on seroprevalence often differ in respect to available sample material, applied serological test, and sample size, making results difficult to compare. We decided to use meat juice for detection of *T. gondii* antibodies in raccoons because this material is more easily accessible from raccoon carcasses than blood and, therefore, more suitable in respect of meat inspection as already discussed for other animals (Berger-Schoch et al. 2011). Although it is generally known that antibody concentration in meat juice is lower than in serum, both matrices were shown to correlate well in serological analyses (Wingstrand et al. 1997). Thus, meat juice was successfully used as an adequate matrix for monitoring meat-producing animals for antibodies to various parasites such as *T. gondii* (Berger-Schoch et al. 2011; Gazzonis et al. 2020; Halos et al. 2010; Lundén et al. 2002) or other zoonotic agents such as *Trichinella* sp. (Nöckler et al. 2005). Due to the general lower concentration of antibodies in meat juice, sample dilutions were adjusted accordingly (Halos et al. 2010; Nöckler et al. 2005; Wallander et al. 2015). The manufacturer’s instruction of the applied ID Screen ELISA test on the one hand advices to use a fivefold lower dilution of meat juice samples (1:2 rather than 1:10 as for serum) and on the other hand also contains an adjusted evaluation scheme for result interpretation from meat juice. From this scheme, samples were considered positive at a lower S/P ratio compared to serum.

Several recent studies about the serostatus of *T. gondii* in raccoons were performed on blood instead of meat juice and using modified direct agglutination test (MAT) or direct agglutination test (DAT) instead of ELISA (Dubey et al. 2007; Heddergott et al. 2017; Hwang et al. 2007; Sato et al. 2011). A previous study on raccoons showed indirect hemagglutination test (IHAT) and latex agglutination test (LAT) inferior to MAT in respect of reaction speed and sensitivity (Dubey et al. 1993). Indirect ELISA is considered a suitable method for the detection of antibodies to *T. gondii* in porcine serum and yielded similar or even better results than MAT (Gamble et al. 2005). ELISA results from the present study were compared to previous research considering varying factors like sample size, study site, or applied serological test, which were discussed to impact varying results (Dubey et al. 2021). Sample size may considerably impact the precision of presented seroprevalence. Therefore, confidence intervals should be considered when comparing results from different studies, especially when sample size was low.

The results of this study, examining meat juice of 820 feral raccoons in Germany by ELISA, revealed a total *T. gondii* seroprevalence of 48.5% (95% CI: 45.1–52.0). The only previous survey also applying ELISA on meat juice to detect antibodies of *T. gondii* in raccoons in Germany reported lower seroprevalence of 33.3% but was based on only 12 samples resulting in a broad 95% CI of 9.9–65.1 (Kornacka et al. 2018) which means that the true prevalence of both studies could be in the same range. Comparing only results from the same region (the federal state Mecklenburg-Western Pomerania), also, only a small number of samples was examined in our study yielding similar results of 35.7% (5/14; 95% CI: 16.3–61.2). The observed overall seroprevalence of 48.5% is higher than in Mecklenburg-Western Pomerania because some federal states like Thuringia, Hesse, and Lower Saxony had prevalence above 50%.

For Hesse, another German federal state, previous studies on raccoon blood reported seroprevalence of 26.0% by IHAT (13/50, no CI indicated) (Gey 1998), 36,4% (8/22; CI: 95% 16.3–56.5), and 65.6% (61/93; CI: 95% 27.3–81.2) by MAT (Heddergott et al. 2017; Heddergott and Müller 2020). Compared to other federal states, we also found high prevalence of 53.1% (93/175; CI: 95% 45.8–60.4) in Hesse. This value is not as high as the one reported by Heddergott and Müller (2020) but is based on a larger sample size and, thus, more precise as indicated by the smaller 95% CI. Besides sample size, the differences may be caused by the different examination methods (ELISA versus MAT), the examined sample material (meat juice versus blood), or the different study sites within Hesse. In the present study, seroprevalence of all regions combined was 48.5%, which is higher than 38.3% (166/433) previously reported by Heddergott et al. (2017) for Germany. As shown in Table 3, seroprevalence greatly varies within national borders, an effect which was similarly reported by others (Burridge et al. 1979; Graser 2008; Heddergott et al. 2017; Mitchell et al. 2006; Sato et al. 2011). Within Germany, seroprevalence seems to decrease from west to east (Fig. 2) resulting in low values in the neighbouring countries Poland (13.3%, 2/15; 95% CI: 1.7–40.5) and the Czech Republic (0%, 0/17; 95% CI: 0–19.5) (Kornacka et al. 2018). However, it should be noted that only one limited area was sampled in these countries and only few animals were examined at all.

Notably, the prevalence of 48.5% detected in this study is comparable to reports from specific regions in the USA, where seroprevalence ranges from 46.5 to 59.2% (Dubey et al. 2007; Gerhold et al. 2017; Lindsay et al. 2001; Mitchell et al. 1999), while it was considerably lower in studies from other US regions and Canada (Fredebaugh et al. 2011; Hwang et al. 2007; Smith et al. 1992), further supporting the influence of region on seroprevalence.

Another commonly discussed influencing factor is urbanization. It is assumed that in urban areas, cats are more prevalent than in rural areas and may serve as a source of environmental contamination with oocysts as source of infection for raccoons. For example, a high seroprevalence of 84.4% was found in raccoons in an urban area of Northern Virginia (Hancock et al. 2005). However, there is inconsistent data to either support or reject that hypothesis. In our study, we stratified sampled animals in regard to the degree of urbanization into three groups based on the European classification scheme (Eurostat 2011) rather than dividing into urban and rural. However, there was no correlation between the degree of urbanization and seroprevalence, which is consistent with the results of Heddergott et al. (2017). Moreover, Graser (2008) and Heddergott and Müller (2020) found lower seroprevalence in raccoons in urban than in rural areas.

There is also ongoing discussion on a correlation between high seroprevalence in raccoons and a high cat density, in general. Hancock et al. (2005) discussed the high density of cats in the sampling area and their oocyst excretion in relation to the high prevalence in their study. This hypothesis is supported by low seroprevalence values in Japan where cat populations are low. Sato et al. (2011), Matoba et al. (2002), and Yamaguchi et al. (2015) detected seroprevalence values of only 9.4–13.7% in raccoons. This might be due to the used LAT method but more likely because of the virtual absence of wild felids in Japan, except for the leopard cat (*Prionailurus bengalensis*), which is found only on the islands Tsushima and Iriomote (Macdonald et al. 2010). Additionally, prevalence of *T. gondii* in domestic cats is similarly low in Japan (9.8%) (Maruyama et al. 2003). Yamaguchi et al. (2015) found a significant higher prevalence in raccoons living near rivers and sharing environment with feral domestic cats. Facilitated by a higher cat density in urban areas, Heddergott and Müller (2020) also discussed that raccoons and cats could get in closer contact in urban areas sharing feeding sites. In other species, such as domestic ruminants in the Mediterranean ecosystem, the presence of cats has been found to be one of the main risk factors for *T. gondii* infection (Almería et al. 2018). Thus, degree of urbanization itself may not be a risk factor to relate *T. gondii* seroprevalence to but rather depends on cat density.

Age was generally reported to be a risk factor for *T. gondii* infection because of the higher chance for contact to the parasite with increasing living time (Jones et al. 2001; Wilking et al. 2016). This was also found in several studies for raccoons (Burridge et al. 1979; Graser 2008; Hill et al. 1998; Hwang et al. 2007; Mitchell et al. 1999; Smith et al. 1992). However, we did not observe a significant difference in seroprevalence between adult and juvenile raccoons, which is in line with Heddergott et al. (2017) as well as Heddergott and Müller (2020), and may be due to the very low number of juveniles (17) compared to adults (801). This imbalance can be explained by these samples originating from a fur producing company, for which juveniles are rarely used and, therefore, hunters almost only provide adult animals. Since we only stratified into two age categories based on dentition status, weight might be a better indicator for already elapsed lifetime of the animals. Although there are limitations like individual food shortage or disease, raccoon weight generally increases with age (Gehrt and Fritzell 1999) and may, therefore, be used as an indicator for age as well as for food intake. Fur impacting disease like canine distemper or prolonged food restriction can be excluded for animals used in the present study since they did not present adverse fur condition or poor general condition. Weight showed a significant influence on serostatus, with heavier animals being more likely seropositive (OR: 1.783). This finding is consistent with other studies analysing raccoon weight as well in Germany (Heddergott et al. 2017; Heddergott and Müller 2020) and Japan (Sato et al. 2011).

For raccoons, sex of the animals also seems to be a potential risk factor. We observed significantly more seropositive males than females. This is comparable with early observations of Burridge et al. (1979). More recently, Hwang et al. (2007) noted that adult males are more often positive than juveniles of both sexes and discussed larger home ranges and a higher mobility as probable reasons. Furthermore, males have a lower need for safety, they sleep in more risky places, and have a larger range of motion than females (Michler 2016). As a result, they also have a different diet compared to female animals, which Engelmann et al. (2011) confirmed by excrement examinations. Together, this may result in an increased risk of exposure to *T. gondii* for males, explaining the observed result.

The noted significantly lower prevalence in autumn compared to spring was also reported in previous studies (Hill et al. 1998; Mitchell et al. 1999). Moreover, Hill et al. (1998) found a correlation between season and age of the animals, with large number of negative juveniles entering the population during the period between spring and autumn. Therefore, a large amount of young and, thus, less likely infected animals are present in autumn compared to spring. Additionally, the diet of free-ranging raccoons greatly varies depending on season and food supply. During autumn, raccoons are more actively feeding on vegetation and crops, while the primary diet in spring is of animals source comprising vertebrates as well as invertebrates (Engelmann et al. 2011). These raccoons’ prey animals are known to transmit *T. gondii* through tissue cysts or oocysts, respectively (Dubey 2021).

The observed high seroprevalence indicates that *T. gondii* is widespread in the German raccoon population and that they might be a relevant source of human *T. gondii* infection during consumption of raccoon meat or fur production handling. Future studies focusing on methods for direct detection of the parasite in meat should be performed to better assess the potential public health risk.

## Data Availability

All supplementary and raw data can be provided on request.
